# Genome-scale metabolic modelling enables deciphering ethanol metabolism via the acrylate pathway in the propionate-producer* Anaerotignum neopropionicum*

**DOI:** 10.1186/s12934-022-01841-1

**Published:** 2022-06-16

**Authors:** Sara Benito-Vaquerizo, Ivette Parera Olm, Thijs de Vroet, Peter J. Schaap, Diana Z. Sousa, Vitor A. P. Martins dos Santos, Maria Suarez-Diez

**Affiliations:** 1 Laboratory of Systems and Synthetic Biology, Wageningen University & Research, Stippeneng 4, Wageningen, 6708WE The Netherlands; 2 Laboratory of Microbiology, Wageningen University & Research, Stippeneng 4, Wageningen, 6708WE The Netherlands; 3 Centre for Living Technologies, Alliance TU/e, WUR, UU, UMC Utrecht, Vening Meinesz building C, Princetonlaan 6, Utrecht, 3584 CB The Netherlands; 4 Bioprocess Engineering, Wageningen University & Research, Droevendaalsesteeg 1, 6708 PB Wageningen, The Netherlands

**Keywords:** Genome-scale, constraint-based metabolic model, Dynamic flux balance analysis, *Anaerotignum neopropionicum*, Acrylate pathway, Ethanol metabolism, Propionic acid

## Abstract

**Background:**

Microbial production of propionate from diluted streams of ethanol (e.g., deriving from syngas fermentation) is a sustainable alternative to the petrochemical production route. Yet, few ethanol-fermenting propionigenic bacteria are known, and understanding of their metabolism is limited. *Anaerotignum neopropionicum* is a propionate-producing bacterium that uses the acrylate pathway to ferment ethanol and CO_2_ to propionate and acetate. In this work, we used computational and experimental methods to study the metabolism of *A. neopropionicum* and, in particular, the pathway for conversion of ethanol into propionate.

**Results:**

Our work describes iANEO_SB607, the first genome-scale metabolic model (GEM) of *A. neopropionicum*. The model was built combining the use of automatic tools with an extensive manual curation process, and it was validated with experimental data from this and published studies. The model predicted growth of *A. neopropionicum* on ethanol, lactate, sugars and amino acids, matching observed phenotypes. In addition, the model was used to implement a dynamic flux balance analysis (dFBA) approach that accurately predicted the fermentation profile of *A. neopropionicum* during batch growth on ethanol. A systematic analysis of the metabolism of *A. neopropionicum* combined with model simulations shed light into the mechanism of ethanol fermentation via the acrylate pathway, and revealed the presence of the electron-transferring complexes NADH-dependent reduced ferredoxin:NADP^+^ oxidoreductase (Nfn) and acryloyl-CoA reductase-EtfAB, identified for the first time in this bacterium.

**Conclusions:**

The realisation of the GEM iANEO_SB607 is a stepping stone towards the understanding of the metabolism of the propionate-producer *A. neopropionicum*. With it, we have gained insight into the functioning of the acrylate pathway and energetic aspects of the cell, with focus on the fermentation of ethanol. Overall, this study provides a basis to further exploit the potential of propionigenic bacteria as microbial cell factories.

**Supplementary Information:**

The online version contains supplementary material available at 10.1186/s12934-022-01841-1.

## Background

Propionic acid is a naturally-occurring carboxylic acid produced by propionigenic bacteria as end-product of their anaerobic metabolism. It is an important intermediate in anaerobic fermentative processes such as those occurring in the human gut, anaerobic digesters and cheese production. It is also an essential platform chemical in the manufacture of cellulose-derived plastics, cosmetics and pharmaceuticals and, due to its antimicrobial properties, it can be used as food preservative [1, 2]. At present, industrial production of propionic acid is based on petrochemical processes, but efforts are being made to develop sustainable production platforms based on the use of propionigenic bacteria as biocatalysts [1, 2]. Microbial production of propionic acid has been researched for over 150 years, however industrial implementation is still limited mainly due to low productivities, which render such processes economically noncompetitive [1–3]. So far, most approaches have considered strains of the genus *Propionibacterium* - well-studied due to their involvement in cheese production [2] -, and have focused on the use of sugars as feedstock. However, the chemical industry is increasingly required to rely on the use of non-conventional, inexpensive raw materials to minimize its carbon footprint [4]. Ethanol, a low-priced common end-product of many fermentations, is regarded as one of such feedstocks [4, 5]. Moreover, ethanol can be synthesised from CO, CO_2_ and H_2_ (syngas) by acetogenic bacteria. Syngas-to-ethanol fermentation technology has been deployed at large scale, and recent advances are expected to accelerate its development in the years to come [6–8].

*Anaerotignum neopropionicum*, formerly *Clostridium neopropionicum* [9], was the first representative of the ethanol-fermenting, propionate-producing bacteria. It was isolated in 1982 from an anaerobic digester treating wastewater from vegetable cannery [10]. The ability of converting ethanol to propionate is shared with only three other microbial species: the closest relative *Anaerotignum propionicum* [11] (formerly, *Clostridium propionicum* [9]), the sulphate-reducing bacterium *Desulfobulbus propionicus* [12, 13], and *Pelobacter propionicus* [14]. In these four microorganisms, ethanol oxidation to propionate occurs in the presence of CO_2_ with concomitant production of acetate, according to the theoretical Eq. 1. This ability of propionigenic bacteria could be exploited to upgrade dilute ethanol streams from beer production or syngas fermentation, among others. For example, Moreira et al. showed that co-cultures of acetogens and ethanol-consuming propionigenic bacteria can convert syngas into propionate [15]. In their study, the acetogen *Acetobacterium wieringae* was co-cultivated with *A. neopropionicum*; *A. wieringae* converted CO to ethanol, which was used by *A. neopropionicum* to produce propionate.

1 $$\begin{aligned} 3 CH_3CH_2OH + 2 CO_2 \leftrightarrow 2 CH_3CH_2COO^- + CH_3COO^- + 3H^+ + H_2O, \end{aligned}$$

$$\Delta G^o = -124 {kJ}.$$

Two main pathways have been described for the fermentative production of propionic acid in bacteria: the methylmalonyl-CoA (also termed succinate pathway or Wood-Werkman cycle) and the acrylate pathway [1, 16]. Most of the known propionigenic bacteria, including strains of the genera *Propionibacterium* and *Cutibacterium*, use the methylmalonyl-CoA pathway for growth. The acrylate pathway is mostly found within members of the phylum Firmicutes [16]. Sugars and lactate are common substrates for these pathways. Ethanol fermenters *D. propionicus* and *P. propionicus* use the methylmalonyl-CoA pathway [13, 14], whereas *A. neopropionicum* and *A. propionicum* use the acrylate pathway [17].

To fully exploit the potential of microorganisms for biotechnological applications, it is fundamental to understand their metabolism and cellular processes. Genome-scale metabolic models (GEMs) and their analysis with COnstraint-Based Reconstruction and Analysis (COBRA) methods [18] have become indispensable tools in this regard [19, 20]. Flux balance analysis (FBA) is often used as the mathematical approach to explore the intracellular fluxes of GEMs under steady-state conditions (e.g., in chemostat cultivations) [21]. FBA can be extended to dynamic FBA (dFBA), which simulates the time-step evolution of individual steady-states that take place in time-varying processes, such as batch and fed-batch cultures [22]. A wide range of GEMs have been successfully implemented to unravel novel metabolic features of microorganisms, guide experimental design or improve bioprocess operation in mono- and co-cultivation. For instance, the reconstruction of the first GEM of *Clostridium ljungdahlii* (iHN637) demonstrated the essential role of flavin-based electron bifurcation in energy conservation during autotrophic growth [23]. FBA enabled the estimation of intracellular metabolic fluxes in the GEM of the acetogen *Clostridium autoethanogenum* (iCLAU786), helping to understand the effects of CO supplementation on CO_2_/H_2_-growing cultures [24]. A multi-species GEM was recently developed that described a syngas-fermenting co-culture composed of *C. autoethanogenum* and *Clostridium kluyveri*; the model provided valuable insight into the microbial interactions between the two microorganisms and predicted strategies for enhanced production of the end products butyrate and hexanoate [25].

Many propionigenic bacteria have been sequenced to date [26–31], including the ethanol fermenters *D. propionicus* [32], *P. propionicus* [33], *A. propionicum* [29] and *A. neopropionicum* [31]. This has enabled the reconstruction of GEMs of some of these species. All GEMs of propionigenic bacteria published to date concern strains that harbour the methylmalonyl-CoA pathway. One of these works described the reconstruction of five *Propionibacterium freudenreichii* species using pan-genome guided metabolic analysis [34]. Navone et. al used the *Propionibacterium* subsp. *shermanii* and the pan-*Propionibacterium* GEMs to guide genetic engineering strategies for increased propionic acid production [35]. Sun et. al developed a constrained-based GEM of *P. propionicus* and validated fermentative growth of this strain on ethanol [36].

Here we describe iANEO_SB607, the first GEM of *A. neopropionicum* and the first to model the acrylate pathway in a propionigenic microorganism. The model was reconstructed using automatic tools followed by an extensive manual curation, which led us to the identification of electron-transferring enzymes involved in the acrylate pathway, cofactor regeneration and energy conservation. In addition, a physiological characterisation of *A. neopropionicum* in batch cultures was performed to validate and complement the reconstruction of the model. FBA was used to assess growth phenotypes on several carbon sources, and dFBA was applied to simulate batch growth of *A. neopropionicum* on ethanol, and ethanol plus acetate. The combination of in-depth modelling and experimentation has enabled us to examine in detail the metabolism of ethanol fermentation in this bacterium and to address pre-existing ambiguities.

## Materials and methods

### Reconstruction of the GEM iANEO_SB607

The genome-scale metabolic network of *A. neopropionicum* was reconstructed in four main steps. First, the genome sequence of *A. neopropionicum* DSM 3847^T^ (GCA$\_$001571775.1) [31] was retrieved from the European Nucleotide Archive in FASTA format and was annotated using RAST [37]. An additional re-annotation was carried out using eggNOG-mapper [38]. The annotation file can be found in the public Gitlab repository: https://gitlab.com/wurssb/Modelling/Anaerotignum_neopropionicum. The second step was the generation of the draft model using ModelSEED [39]. For this, the RAST annotation file was imported into ModelSEED and a Gram-positive template was chosen to reproduce growth on rich medium. The draft model was downloaded in table format and SBML format. The third step consisted on the manual curation and refinement of the draft model. Every reaction entry was analysed individually and modifications were made on the table format file. Specifically, (i) unbalanced reactions were corrected based on charged formulas with the corresponding addition/deletion of H^+^ or H_2_O molecules; (ii) reaction direction was adjusted using eQuilibrator [40]. Reactions were considered reversible if the change in Gibbs free energy was between -30 and 30 kJ mol^-1^ at standard conditions for reactants/products, pH 7.3 and ionic strength 0.1 M. In cases where eQuilibrator did not retrieve information for a specific reaction, reaction direction was adjusted based on information from MetaCyc [41] and BIGG [42] databases. (iii) EC numbers were corrected or inserted for every reaction based on information from KEGG [43] and MetaCyc [41]. (iv) The original genes in Patric format [44] were replaced by the locus tag format (’CLNEO_XXXXX’) found in UniProt [45] and BRENDA [46] databases. The re-annotation file was used to identify potential gene(s) associated to reactions that lacked a gene in the original RAST annotation. (v) The final step consisted of gap-filling, where reactions were added or removed to reproduce known or observed phenotypes. Gap-filling was done combining a computational and a manual approach: an automatic gap-filling process was run using the KBase pipeline[47], while the manual curation was based on experimental data obtained in this study and published works. The final model, iANEO_SB607, can be found in the git repository in Table format, json and SBML L3V1 [48] standardization. Furthermore, the different versions together with a Memote and FROG report (https://www.ebi.ac.uk/biomodels/curation/fbc) were combined in an OMEX archive file [49] deposited in BioModels [50] and assigned the identifier MODEL2201310001.

#### Generation of the biomass synthesis reaction and sensitivity analysis

The biomass reaction of *A. neopropionicum* was adapted from the biomass reactions of *Clostridium beijerinckii* (GEM iCM925 [51]) and *C. autoethanogenum* (GEM iCLAU786 [52]). The composition of the main building blocks was maintained but, based on the protocol of Thiele and Palsson [53], protons were stoichiometrically added to the hydrolysis part of the biomass synthesis reaction. Protons were also added to the reactions of DNA, RNA, proteins, teichoic acids and peptidoglycans synthesis in line with the ATP associated to polimerization. The DNA composition was determined based on the GC content of the genome of *A. neopropionicum* and it was adjusted in the reaction associated to the biosynthesis of DNA. The fatty acids composition was adjusted based on reported experimental data for *A. neopropionicum* [9].

A sensitivity analysis was performed by modifying the content of proteins, phospholipids (plipids) and cell wall components, considering cell wall components as the sum of teichoic acid, peptidoglycans and carbohydrates composition. The rest of components—DNA, RNA and trace- were kept fixed, as together they only represent 10% of the biomass. The composition of proteins and plipids was randomly selected within ± 10% of their original value. In this way, the total cell wall components composition was calculated following equation 2.

2 $$\begin{aligned} Cell\; wall\; components\; = 1-protein-plipids-(DNA+RNA+trace) \end{aligned}$$

Consecutively, the value of each cell component was distributed within teichoic acid, peptidoglycans and carbohydrates following the same proportion as they had in the original biomass synthesis reaction. For each randomly selected value, a new biomass synthesis reaction was obtained. This new biomass synthesis reaction was maximised as the objective function using FBA in COBRApy [54] maintaining fixed ethanol and CO_2_ uptake rates. We repeated this process 1000 times, so that we obtained 1000 different biomass synthesis reactions. The composition of the cell wall components, proteins and phospholipids was stored for each biomass synthesis reaction, together with the growth rate, and acetate and propionate production rates. The obtained growth rate, acetate and propionate production rates were normalised with respect the original values and were plotted against each biomass building block (Additional file 1: Fig. S1) Additionally, we also studied the effect of varying GAM on the growth rate. In this analysis, the original fractions of the biomass components shown in equation 2 were maintained, and we randomly selected different GAM values within ±20% of the original value. We repeated this process 1000 times and calculated the growth rate for each GAM value. The obtained growth rate was normalised with respect the original growth rate and was plotted against GAM (Additional file 1: Fig. S2; git repository).

### Model simulations at steady-state

The model was qualitatively validated by assessing growth capabilities and product profile on several carbon sources in steady-state. Model simulations were done using COBRApy, version 0.24.0 [54], and Python 3.6.9. The maximum empirical ethanol uptake rate across cultivations was 30 to 40 mmol g_DW_^-1^ h^-1^ (see Quantitative assessment of iANEO_SB607 through dFBA). Based on this, the lower bound of the substrate uptake rate per time point was constrained to 30 mmol g_DW_^-1^ h^-1^ to assess growth on a single carbon source, and to 30 mmol g_DW_^-1^ h^-1^ in total to assess growth on more than one carbon source, unless specified otherwise. The biomass synthesis reaction was used as the objective function. Growth was considered when the growth rate was higher than 0.0001 h^-1^. To better explore the solution space, the fluxes compatible with the applied constraints were sampled using the sample function with the ’achr’ method in the flux_analysis submodule of COBRApy [55]. The lower bound of the biomass synthesis reaction was constrained to be at least 99% of the maximum growth rate calculated by FBA. Presented results are the average and standard deviation based on 5000 iterations generated at each condition.

### Dynamic flux balance analysis simulations

The reconstructed GEM iANEO_SB607 was subjected to dFBA to simulate batch growth of *A. neopropionicum* on ethanol and ethanol plus acetate. Model simulations were done using COBRApy, version 0.24.0 [54], IBM ILOG CPLEX 128, and Python 3.6.9 (see git repository). The maximum uptake rate, maximum growth rate and initial substrate and biomass concentration, obtained from batch cultivations, were used as model inputs. To constrain the feasible flux space, ethanol uptake was specified to follow a Michaelis-Menten-like kinetics (Eq. 3) with parameters q_Si,max_ and K_m,i_:

3 $$\begin{aligned} q_{Si} = \frac{q_{Si,max} S_{i}}{K_{m,i} + S_{i}} \end{aligned}$$

where q_Si_ is the uptake rate of substrate *i* (mmol g_DW_$^{-1}$ h$^{-1}$); q_Si,max_ is the maximum uptake rate of substrate *i* (mmol g_DW_$^{-1}$ h$^{-1}$); K_m,i_ is the Michaelis-Menten constant (mM) for substrate *i* and S_i_ is the concentration of substrate *i* (mM). K_m,i_ was determined based on experimental data and model fitting (Additional file 1: Table S1). q_Si,max_ was calculated from experimental data of batch fermentations. Concentrations of substrates, products and biomass over time were determined as follows. First, the Vs_i_ was calculated using Eq. 3 for each given time step and the defined initial concentrations. Then, FBA was applied under those constraints to compute the fluxes at maximum growth rate. After that, the following ordinary differential equations (ODE) were solved:

4 $$\begin{aligned} \frac{dX_{i}}{dt}= {\mu}{X_{i}}, \end{aligned}$$

5 $$\begin{aligned} \frac{dS_{i}}{dt}= {q_{Si}}{X_{i}}, \end{aligned}$$

6 $$\begin{aligned} \frac{dP_{j}}{dt}= {q_{Pj}}{X_{i}}, \end{aligned}$$

where X_i_ is the biomass concentration (g L^-1^); $\mu$ is the specific growth rate (h^-1^); S_i_ is the concentration of substrate *i* (mM); q_Si_ is the uptake rate of substrate *i* (mmol g_DW_$^{-1}$ h$^{-1}$); q_Pj_ is the production rate of product *j* (mmol g_DW_$^{-1}$ h$^{-1}$), and P_j_ is the concentration of product *j* (mM). Equations 4,5 and 6 were used to calculate X, S_i_ and P_j_, respectively. S_i_ is used as input to calculate the next state following Eq. 3. The objective function was changed to maximise the ATP generation (“rxn00062_c0”) once the model became infeasible due to the low concentration of ethanol. For each time step, the concentration of biomass, substrate and products was computed and the calculated values were stored and plotted.

### Experimental batch fermentation data

#### Cultivation conditions

*A. neopropionicum* DSM 3847^T^ was obtained from the German Collection of Microorganisms and Cell Cultures (DSMZ, Braunschweig, Germay). Batch fermentations were done in 117 mL serum bottles containing 50 mL medium with the following composition (per litre): 0.9 g NH_4_Cl, 0.3 g NaCl, 0.8 g KCl, 0.2 g KH_2_PO_4_, 0.4 g K_2_HPO_4_, 0.2 MgSO_4_ x 7 H_2_O, 0.04 CaCl_2_ x 2 H_2_O, 3.36 g NaHCO_3_, 10 mL trace element solution from DSM medium 318, 1 mL vitamin solution, 0.5 g yeast extract, 0.3 g Na_2_S x *x* H_2_O (*x*=9-11) as reducing agent and 0.5 mg resazurin as redox indicator. The vitamin solution contained (per liter): 0.5 g pyridoxine, 0.2 g thiamine, 0.2 g nicotinic acid, 0.1 g p-aminobenzoate, 0.1 g riboflavin, 0.1 g pantothenic acid, 0.1 g cobalamin, 0.05 g folic acid, 0.05 g thioctic acid and 0.02 g biotin. The headspace of the bottles was filled with a gas mixture of N_2_/CO_2_ (80:20 % v/v; 170 kPa). To test growth in the presence of H_2_, the headspace of bottles was filled instead with a gas mixture of H_2_/CO_2_/N_2_ (10:20:70 and 80:20:0 % v/v; 170 kPa). Growth was assessed on the following substrates: ethanol, lactate, glucose and xylose, at an initial concentration of 25 mM. Where indicated, acetate (10 or 25 mM) was added to ethanol-fed cultures. The pH of the medium was 7.1 - 7.2. Cultures were incubated at 30^o^C statically.

#### Analytical techniques

Liquid and headspace samples were taken periodically over the course of batch fermentations and analysed for biomass, substrate and product concentrations. Biomass growth was measured by optical density at 600 nm (OD_600_). Biomass concentration (mg_CDW_ L^-1^) was estimated from OD_600_ measurements using the correlation: mg_CDW_ L^-1^ = (OD_600_ - 0.016)/0.0032, which was experimentally determined from *A. neopropionicum* cultures grown on ethanol. Concentrations of soluble compounds in the supernatant of liquid samples were determined using high-pressure liquid chromatography (HPLC) (LC-2030C Plus, Shimadzu, USA). The HPLC was equipped with a Shodex SH1821 column operated at 65^o^C. A solution of 0.1 N H_2_SO_4_ was used as mobile phase, at a flowrate of 1 mL/min. Detection was done via a refractive index detector. Concentrations below 0.2 mM could not be accurately quantified and are considered traces. Concentrations of gases in headspace samples were determined via gas chromatography (GC) (Compact GC 4.0, Global Analyser Solutions, The Netherlands). To analyse H_2_, a Molsieve 5A column operated at 140^o^C coupled to a Carboxen 1010 column was used. CO_2_ was analysed in a RT-Q-BOND column at 60^o^C.

## Results

### Reconstruction of iANEO_SB607, the first GEM of *A. neopropionicum*

A draft model of the metabolism of *A. neopropionicum* was developed by automatic reconstruction using the publicly available genome sequence of the microorganism (DDBJ/EMBL/GenBank accession number: LRVM00000000; [31]). The draft model comprised 491 genes, 855 metabolites and 907 reactions. This preliminary model predicted growth only on rich medium supplemented with amino acids and biomass precursors, and it did not predict the production of propionate and acetate. We performed an extensive manual curation process that resulted in the deletion, modification or addition of reactions, metabolites and genes (see git repository). The final model, iANEO_SB607, comprises 607 genes, 815 metabolites and 932 reactions (Table 1). This is the first GEM of the propionigenic bacterium *A. neopropionicum*.

**Table 1:** Composition of iANEO_SB607

Features	Amount
Genes	607
Metabolites	815
Intracellular metabolites	742
Extracellular metabolites	73
Reactions	932
Metabolic reactions	771
Transport reactions	88
Exchange reactions	73
Reactions associated with genes	733
Reactions non-associated with genes	199

**Fig. 1:**
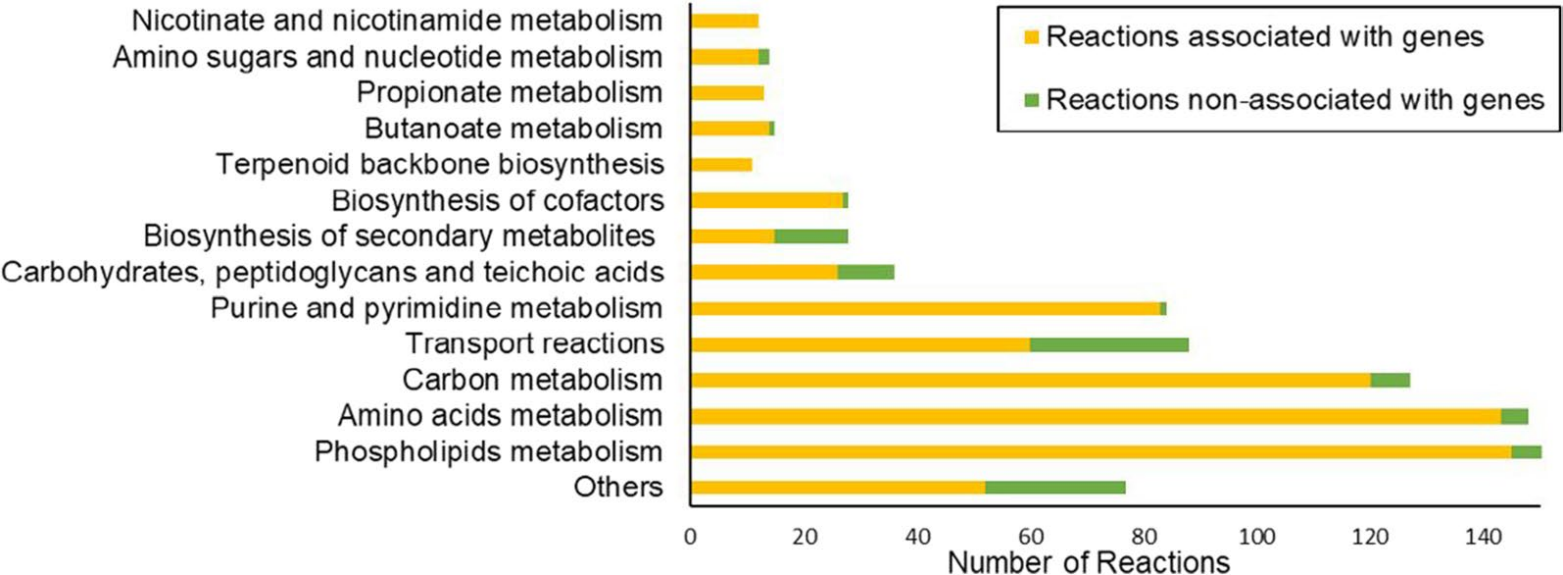
Distribution of the reactions of the iANEO_SB607 model within cellular subsystems

Two compartments are recognised in the model: the intracellular compartment (id: ’c0’) and the extracellular compartment (id: ’e0’). Metabolites are assigned to either one of the compartments. Reactions are classified as metabolic reactions, transport reactions and exchange reactions. Metabolic reactions describe the biochemical conversion of metabolites within the intracellular compartment. Transport reactions describe the transport of metabolites across the intracellular and extracellular compartments. Exchange reactions simulate the excretion of metabolites outside the cell or the uptake of metabolites into the cell. Reactions are distributed within cell subsystems (Fig. 1), except exchange reactions. The model also includes reactions involved in the production of acetate, propionate, butyrate, propanol, isobutyrate and isovalerate. Approximately 80 % of reactions could be associated to genes present in the genome of *A. neopropionicum*. The remaining 20 % of reactions are not associated with genes. Half of these reactions are mostly exchange reactions and diffusion transport reactions. The other half are spontaneous reactions or gap-filled reactions describing, in a summarised manner, the biosynthesis of biomass building blocks (e.g., lipids, carbohydrates).

### Sensitivity analysis of the biomass synthesis reaction

The constructed biomass synthesis reaction (BIOMASS_Aneopro_w_GAM) accounts for the production of DNA, RNA, proteins, peptidoglycans, phospholipids, teichoic acids and trace, and it is normalised to 1 gram per mmol. It also includes the growth-associated ATP maintenance (GAM) as an hydrolysis reaction, and the non-growth associated ATP maintenance (NGAM) as a reaction of ATP phosphohydrolase (rxn00062_c0). GAM was assumed to be 40 mmol ATP/g_DW_, as in the GEM of *C. acetobutylicum* [56]. The lower bound of this reaction was constrained to a rate of 8.4 mmol ATP g_DW_^-1^ h^-1^, an estimation based on the models of *C. beijerinckii* [51] and *C. autoethanogenum* [52].

Since the biomass synthesis reaction of *A. neopropionicum* was developed based on these two other species, we performed a sensitivity analysis to test its robustness. The analysis showed the effect of modifying the proportion of the main biomass components from the biomass synthesis reaction on model predictions (i.e., growth and production rates). In all scenarios tested, growth and production rates remained virtually unaffected (Additional file 1: Fig. S1). The largest deviation of the growth rate, acetate and propionate production rates were ± 0.0005 h^-1^, ± 0.005 mmol g_DW_^-1^ h^-1^ and ± 0.0025 mmol g_DW_^-1^ h^-1^, respectively, which are negligible as they only represent 3, 0.025 and 0.025 %, respectively. The effect of varying other biomass components -DNA, RNA and trace- was also considered negligible given that they represent a minor fraction of the biomass (10%). The growth rate was slightly more affected when GAM was changed. The largest deviation was ± 0.00175 h^-1^, which corresponds to 10.8 % difference compared to the original growth rate. The biomass synthesis reaction was therefore considered a reliable representation of the biomass composition of *A. neopropionicum*.

### Quality of the GEM iANEO_SB607

The quality of the iANEO_SB607 model was evaluated using the SBML validator [57] and the test suite Memote [58]. Additionally, we have run a FROG analysis to verify the reproducibility of the model. The GEM was correctly defined in SBML format, level 3, version 1. The GEM obtained an overall Memote score of 72 %. All metabolites, reactions and genes were fully annotated. The annotation per database of reactions and metabolites scored 83 %, however the annotation per database of genes scored a much lower value, 33 %. Reactions are mass and charge balanced, except for reactions associated to the synthesis of biomass precursors. The model does not have infeasible cycles and all metabolites are connected. However, the model is only partly consistent (55 % scoring); this is due to the creation of metabolites to account for biomass precursors. These metabolites (e.g., RNA) lack a defined formula or a correct charge and, thus, their associated reactions are considered stoichiometrically inconsistent, decreasing the global consistency score. Memote identifies 102 metabolites that can only be consumed or produced, resulting in 422 blocked reactions in the model under the restrictive constraints. When the model does not have constraints, FVA analysis finds 354 blocked reactions,which is in line with the average % of blocked reactions in GEMs (20-40%) [59].

### Qualitative assessment of iANEO_SB607 through analysis of growth phenotypes

The iANEO_SB607 model was qualitatively validated by assessing growth of *A. neopropionicum* on several carbon sources and contrasting the results with experimental data. Model predictions matched most of the growth phenotypes observed in cultivation experiments from this and previous studies (Table 2; full data is available in the git repository and Additional file 1: Table S2).

**Table 2:** Growth phenotypes of *A. neopropionicum* on different substrates, predicted by the iANEO_SB607 model and observed in experiments from this and previous studies

Substrates	iANEO_SB607	This study (exp.)	[17]	[9]	[15]
Ethanol	+	+	+	+	+
Ethanol and acetate	+	+	+	ND	ND
Ethanol and alanine	+$^{[a]}$	ND	ND	ND	+
Ethanol and serine	+$^{[b]}$	ND	ND	ND	+
Pyruvate	+	ND	+	+	ND
D-Lactate	+	+	+	w$^{[c]}$	ND
D-Glucose	+	+	+	-	ND
Xylose	+	+	+	+	ND
L-Threonine	+	ND	+	+	ND
L-serine	+	ND	+	+	ND
L-Alanine	+	ND	+	+	ND
D-Alanine	+	ND	+	ND	ND
L-Valine	–	ND	–	w	ND
L-Leucine	–	ND	ND	w	ND
L-Isoleucine	–	ND	ND	w	ND
Lysine	–	ND	–	ND	ND
L-Proline	-	ND	–	–	ND

+ Positive, − negative, *w* weakly positive,* ND* no available data

^a^ L-Alanine

^b^ L-Serine

^c^ (L-D)-Lactate

The model predicts growth of *A. neopropionicum* on ethanol. Growth on xylose and on glucose is also predicted by the model and supported by experimental evidence, with exception of one study, which reported no growth of *A. neopropionicum* on glucose [9]. According to a previous work, *A. neopropionicum* can also grow on D-lactate, but not on L-lactate [17]. In our batch cultivations with DL-lactate as substrate, we repeatedly observed that only $\approx$ 50 % of the substrate was used. The purity of the L- enantiomer in the racemic mixture solution was, according to the manufacturer, 27 - 33 %. This indicates that D-lactate is indeed used by *A. neopropionicum*, but it does not exclude the possibility that L-lactate is also metabolised. Yet, since the latter could not be confirmed, the model considers only the utilisation of D-lactate. The model predicts growth on pyruvate as well as on one pyruvate-derived amino acid, alanine. Serine also supports growth of *A. neopropionicum*, as predicted by the model and observed in cultivation experiments. The model indicates that branched-chain amino acids (valine, leucine and isoleucine) as well as TCA-derived amino acids (lysine and proline), with exception of threonine, are not utilised.

**Fig. 2:**
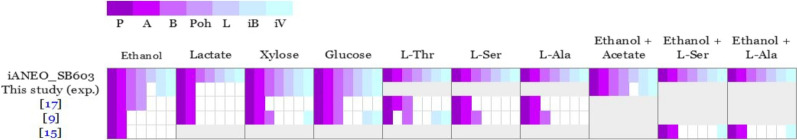
Product profile of the fermentation of different substrates by *A. neopropionicum*, predicted by the GEM iANEO_SB607 and observed in experiments from this and previous studies. P: Propionate; A: acetate; B: butyrate; Poh: propanol; L: lactate; iB: isobutyrate and iV: isovalerate. White spaces indicate the product is not reported produced. Grey areas indicate no available data

Further model validation was performed by assessing the product profile on a number of substrates from which sufficient experimental data was available, specifically: ethanol, lactate, glucose, xylose, L-threonine, L-serine, L-alanine, ethanol plus acetate, ethanol plus L-serine and ethanol plus L-alanine. For all the substrates tested, the model predicted mixed secretion of propionate and acetate, in accordance with experimental evidence (Fig. 2; full data is available in the git repository and Additional file 1: Table S2). Model analysis shows that secretion of product mixture is a requisite for energy generation and redox cofactor regeneration. The involved pathways and their stoichiometry are described in following sections.

Butyrate, propanol, lactate, isobutyrate and isovalerate are also predicted by the model as fermentation products in all cases, albeit in different proportions. Butyrate appears as a minor product in all the simulations and cultivation experiments, except for in the fermentation of L-threonine; in this case, the model predicts butyrate as a major end product, as previously reported [9]. According to model simulations and in agreement with our experimental data, lactate, an intermediate of the acrylate pathway, and propanol are produced in minor amounts. In batch cultivations carried out in this study, isobutyrate and isovalerate were detected as traces with ethanol (plus acetate), glucose or xylose as substrates, but not with lactate. The model predicted both products to be produced as traces with these substrates. Model simulations predicted enhanced production of isobutyrate and isovalerate with ethanol plus L-valine and ethanol plus L-leucine as substrates, respectively (not shown), as observed in one study [9]. The model also predicted the production of isovalerate when L-alanine or L-serine are co-substrates with ethanol, which is in agreement with observations from a recent work [15].

H_2_ was not detected as product in any of the fermentations of *A. neopropionicum* carried out in this study (with substrates: ethanol (plus acetate), lactate, glucose, xylose). In addition, H_2_ was not utilised nor affected the growth or the product profile of *A. neopropionicum* cultures growing on ethanol (Additional file 1: Fig. S3). Previous works reported the same observations [17, 60]. A ferredoxin hydrogenase is annotated in the genome of *A. neopropionicum* (CLNEO_18070; EC 1.12.7.2; model id:’rxn05759_c0’); yet, given the collected evidence, this reaction was blocked in the model.

### Quantitative assessment of iANEO_SB607 through dFBA

The iANEO_SB607 model of *A. neopropionicum* was evaluated quantitatively by simulating the dynamics of batch fermentation using dFBA. Three conditions were considered, with regard to the substrates present: 25 mM ethanol, 25 mM ethanol plus 10 mM acetate, and 25 mM ethanol plus 25 mM acetate. To constrain the model, we used empirical data of ethanol consumption, product formation and cell growth from cultivation experiments. The fermentation profiles obtained by dFBA were contrasted with the experimental data of batch incubations. Across cultivations, carbon balance was 85 - 96 %, not completely closed likely due to the difficulty to accurately quantify CO_2_ and to slight evaporation of ethanol in the bottles, as reported by others [61].

**Fig. 3:**
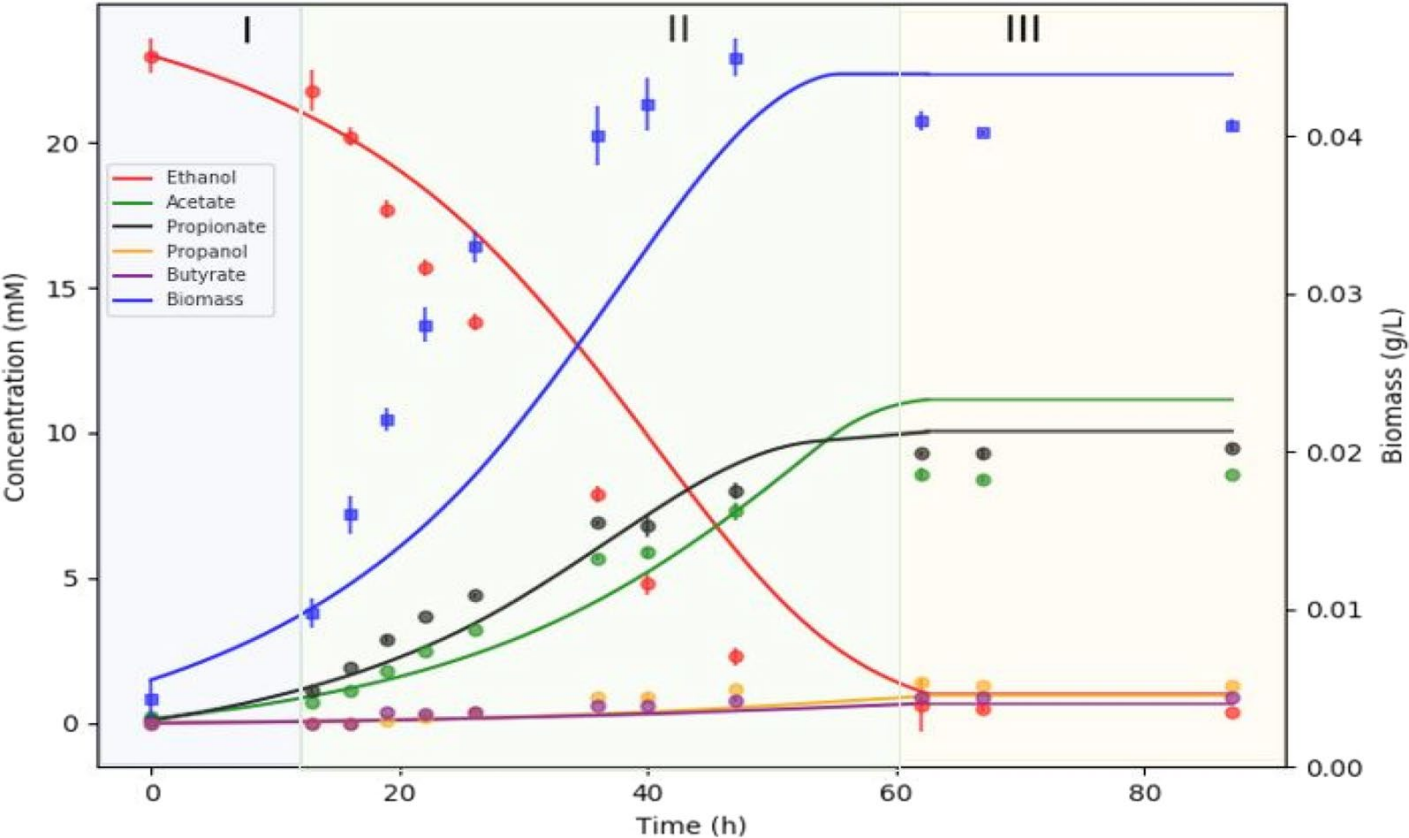
Fermentation of ethanol (25 mM) by *A. neopropionicum* in batch cultivation. Dots indicate experimental data, and solid lines indicate the result of dFBA. Background colours distinguish fermentation phases: lag (blue), exponential (green) and stationary (orange)

For the condition with only ethanol (and CO_2_) as substrate, the time-course data obtained through dFBA accurately reproduced the fermentation profile, with only small deviations (Fig. 3). Exponential growth of *A. neopropionicum* began after a relatively short lag phase of $\approx$ 13 hours. During the exponential phase, ethanol was uptaken (together with CO_2_; not shown) at an empirical maximum consumption rate (q_S,max_) of 36.2 ± 5.5 mmol ethanol g_DW_^-1^ h^-1^. Modeled ethanol consumption fitted the experimental data with a small margin of error. Propionate and acetate were produced simultaneously during the exponential phase, at empirical maximum production rates (q_P,max_ and q_A,max_) of 12.0 ± 0.1 mmol propionate g_DW_^-1^ h^-1^ and 8.6 ± 0.5 mmol acetate g_DW_^-1^ h^-1^, respectively. The production profile of propionate was well predicted by dFBA, estimating a final propionate concentration (10.9 mM) close to the experimental value (9.5 mM). However, dFBA predicted a final concentration of acetate (11.5 mM) moderately higher than experimentally observed (8.6 mM). The empirical maximum specific growth rate of *A. neopropionicum* ($\mu$_max_) was 0.082 ± 0.006 h^-1^ (duplication time = 8.4 h), which was used to constrain the model. In incubations, the biomass concentration peaked (44.7 ± 1.3 mg_DW_ L^-1^) at $\approx$ 47 hours, and decreased afterwards. The simulation predicted a slightly deviated pattern of biomass formation during the exponential phase, and it did not predict the observed drop in the stationary phase. Yet, the predicted maximum biomass concentration (44 mg_DW_ L^-1^) matched the empirical value. Propanol (1.3 mM) and butyrate (1 mM) were detected as minor products in batch incubations; the evolution of both products was predicted correctly by the dFBA simulations. Traces of isobutyrate and isovalerate were also detected and predicted by dFBA (not shown).

**Fig. 4:**
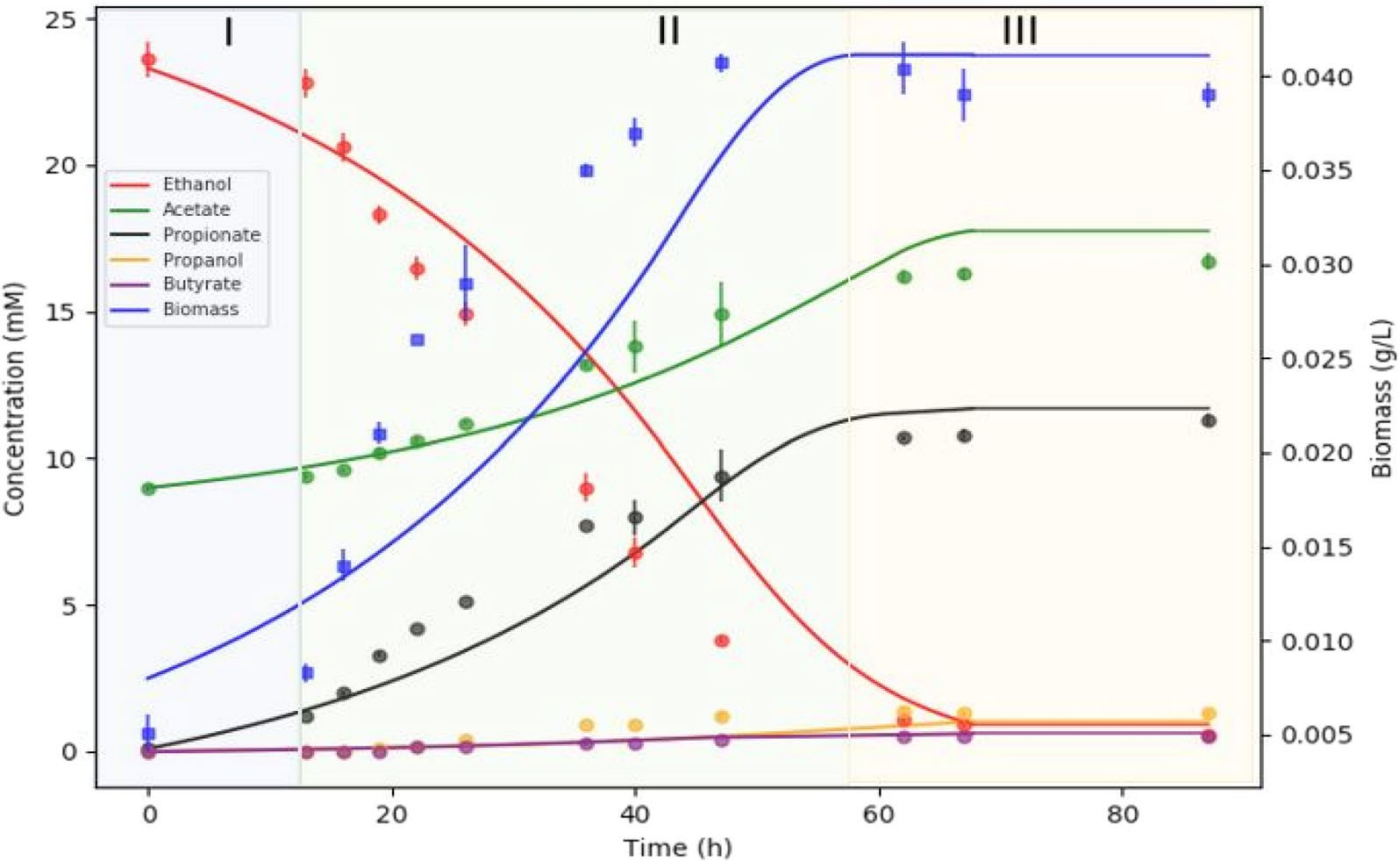
Fermentation of ethanol (25 mM) and acetate (10 mM) by *A. neopropionicum* in batch cultivation. Dots indicate experimental data and solid lines indicate the result of dFBA. Background colours distinguish fermentation phases: lag (blue), exponential (green) and stationary (orange)

To further evaluate the ability of *A. neopropionicum* to upgrade dilute ethanol streams from syngas fermentation, we considered a scenario with ethanol and acetate as co-substrates. Acetate is produced by acetogens as a major product of autotrophic metabolism, and it is therefore found in variable proportions in syngas fermentation effluent. *A. neopropionicum* can utilise acetate in the presence of propanol [10] or ethanol [17] as electron donors. To investigate the effect of acetate as co-substrate on ethanol-fermenting cultures of *A. neopropionicum*, incubations were set up with ethanol (25 mM) and acetate (10 and 25 mM) as susbtrates, and dFBA was used to simulate the dynamics of these fermentations. dFBA reproduced with high accuracy the fermentation profile of incubations containing ethanol plus 10 mM acetate (Fig. 4). In this condition, the observed $\mu$_max_ was 0.098 ± 0.005 h^-1^ (duplication time = 7.1 h); 19 % higher than in the incubations without acetate. However, less biomass was formed in comparison; the maximum biomass concentration was 41.1 ± 0.8 mg_DW_ L^-1^ ($\approx$ 9% lower), which was also predicted by dFBA. The presence of 10 mM acetate also affected the consumption and production rates; ethanol consumption was faster than in the absence of acetate; the q_S,max_ was 43.3 ± 4.3 mmol ethanol g_DW_ h^-1^, a 20 % increase. The q_A,max_ in this condition dropped to 3.1 ± 0.6 mmol acetate g_DW_ h^-1^. The biggest difference was in the q_P,max_, which was 16.4 ± 0.8 mmol propionate g_DW_ h^-1^, a 37 % increase compared to the condition without acetate. The final propionate concentration was also slightly higher, 11.3 mM (vs. 9.5 mM). Here, again, the simulation predicted a similar propionate concentration to the observed value (12.2 mM), and a higher final acetate concentration (18.3 mM) than observed (16.7 mM). The incubations containing 25 mM acetate at the start followed a different trend than the incubations with 10 mM acetate (fermentation profile not shown). In batch bottles, the biomass concentration reached a similar value to that obtained in the condition with 10 mM acetate, but the $\mu$_max_, q_P,max_ and q_A,max_ were similar to the condition without acetate (data not shown). The final propionate concentration was 12.5 mM, the highest of the three conditions tested.

The presence of acetate had an effect on the utilisation of ethanol by *A. neopropionicum*, which is reflected in the fermentation yields. The biomass yield (Y_X/S_) was slightly lower in the presence of both 10 and 25 mM acetate (1.4 g_DW_ mol ethanol^-1^ vs. 1.6 g_DW_ mol ethanol^-1^ when no acetate was present). With acetate present at the start of incubations, more ethanol was invested in propionate production, as indicated by the propionate yields (Y_P/S_, mol mol^-1^), which were 0.33, 0.38 and 0.42 for the conditions with no acetate, 10 mM acetate and 25 mM acetate, respectively. The production of acetate followed the inverse trend; acetate yields (Y_A/S_, mol mol^-1^) were 0.29, 0.18 and 0.06 for the conditions with no acetate, 10 mM acetate and 25 mM acetate, respectively. Similarly, lower yields were obtained for propanol and butyrate when acetate was present (data now shown).

### Ethanol fermentation via the acrylate pathway

**Fig. 5:**
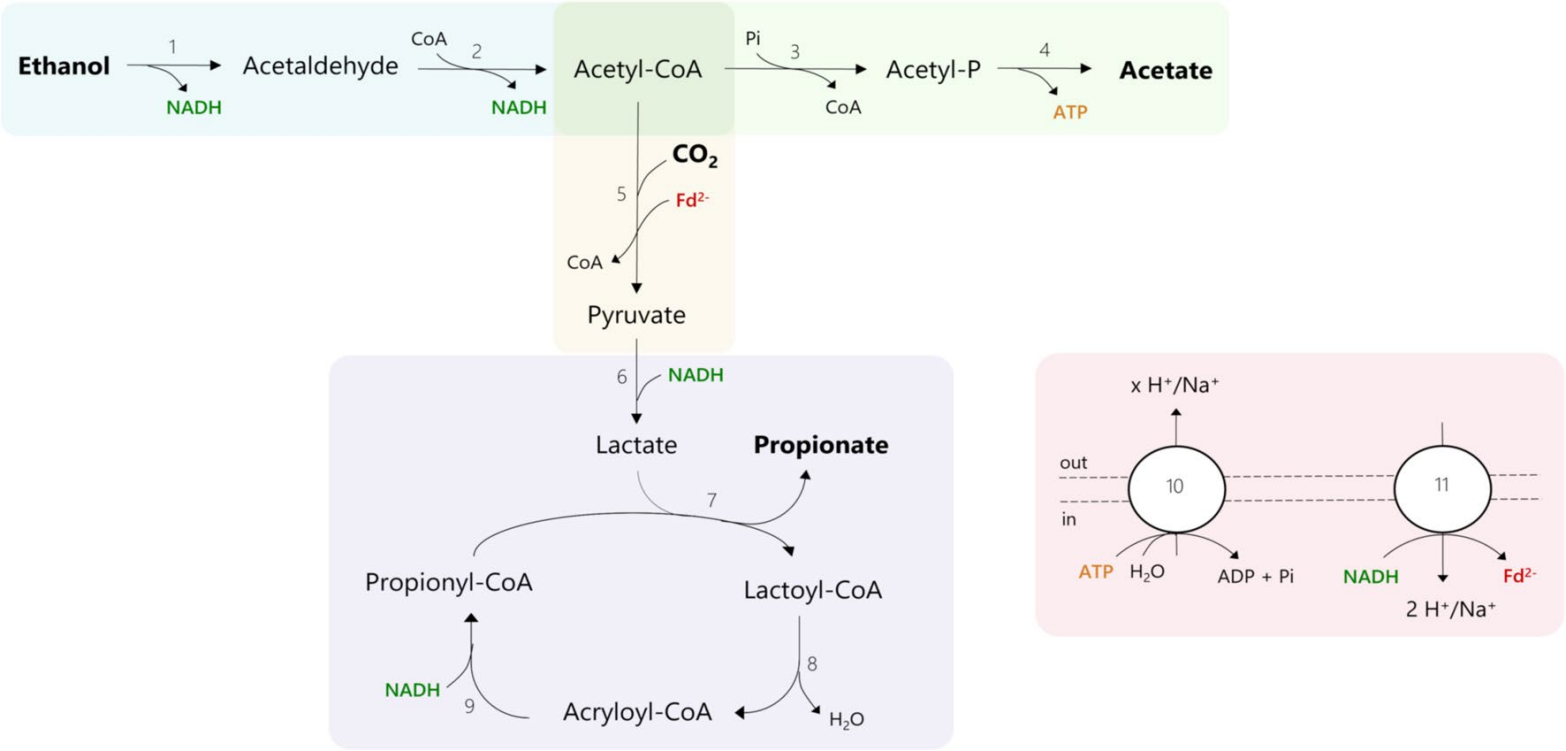
Proposed metabolism of ethanol fermentation to propionate via the acrylate pathway in *A. neopropionicum*. Coloured areas designate the following modules: ethanol oxidation (blue), acetate production (green), pyruvate synthesis (yellow), lactate production and acrylate pathway (purple), redox cofactor regeneration and ATPase (red). Numbers in reactions correspond to the following enzymes and reaction ids in the model: 1,2, aldehyde-alcohol dehydrogenase (rxn00543_c0 and rxn00171_c0); 3, phosphate acetyltransferase (rxn00173_c0); 4, acetate kinase (rxn00225_c0); 5, pyruvate:ferredoxin oxidoreductase (PFOR; rxn05938_c0); 6, NAD-dependent D-lactate dehydrogenase (rxn00500_c0); 7, propionate-CoA:lactoyl-CoA transferase (rxn01056_c0); 8, lactoyl-CoA dehydratase (rxn02123_c0); 9, acryloyl-CoA reductase (rxn40050_c0); 10, ATPase (rxn10042_c0); 11, Rnf complex (Rnf_c0)

The reconstructed iANEO_SB607 model describes the metabolism of ethanol fermentation and propionate production via the acrylate pathway in *A. neopropionicum* (Fig. 5). Model simulations provided new insights into the enzymatic reactions involved in propionate formation, cofactor regeneration and the energy metabolism of the cell.

Ethanol is oxidised to acetyl-CoA via acetaldehyde through alcohol and acetaldehyde dehydrogenases. The genome of *A. neopropionicum* harbours a bifunctional NAD^+^-dependent alcohol-aldehyde dehydrogenase (AdhE; CLNEO_13930) that can catalyse this two-step conversion. According to our model, two other alcohol dehydrogenases, encoded by *adh* (CLNEO_16910) and *adhB* (CLNEO_00480), could also drive the oxidation of ethanol to acetaldehyde. Initially, the model also predicted this reaction to be catalysed by NAD(P)H-dependent butanol dehydrogenase (BdhA), encoded by *bdhA* (CLNEO_09740; rxn00536_c0). However, the well-characterised BdhA of *C. acetobutylicum*, which shares 60.7 % identity with that of *A. neopropionicum*, is known to contribute primarily to butanol production and it is the alcohol dehydrogenase least involved in ethanol metabolism [62]. Thus, we reasoned that BdhA would likely not be involved in ethanol oxidation in *A. neopropionicum* and excluded this reaction from model simulations.

Acetyl-CoA is partly used in the reductive reactions of the metabolism and partly invested in the formation of acetate, an energy-generating step. Acetate is synthesised via phosphate acetyltransferase (Pta; CLNEO_28570) and acetate kinase (Ack; CLNEO_28580), yielding ATP via substrate-level phosphorylation (SLP). In the reductive path, acetyl-CoA is converted to pyruvate through the CO_2_-fixating reaction catalysed by pyruvate:ferredoxin oxidoreductase (PFOR; CLNEO_15240 or CLNEO_19010 or CLNEO_17780 or CLNEO_03040 or CLNEO_04330 or CLNEO_24550). This conversion requires reduced ferredoxin (Fd^2-^) as electron carrier. Our hypothesis, supported by model predictions, is that Fd^2-^ is produced in the Na^+^-translocating ferredoxin:NAD^+^ oxidoreductase (Rnf) complex. The Rnf complex is a membrane-bound respiratory enzyme involved in energy conservation in anaerobic microorganisms [63]. During growth on high-energy substrates, it catalyses the exergonic reduction of NAD^+^ with electrons from Fd^2-^ coupled to the translocation of two cations (H^+^ or Na^+^) across the membrane. The electrochemical potential established by the Rnf complex can then be used by a membrane-bound ATP synthase for energy generation. The Rnf complex can also operate in the reverse direction to produce Fd^2-^ at the expense of ATP [64]. The genome of *A. neopropionicum* harbours a complete *rnf* cluster, composed of the genes *rnfA* (CLNEO_01390), *rnfB* (CLNEO_01400), *rnfC* (CLNEO_01350), *rnfD* (CLNEO_01360), *rnfE* (CLNEO_01380) and *rnfG* (CLNEO_01370). With ethanol as substrate, our assumption is that the Rnf complex of *A. neopropionicum* operates in reverse, generating Fd^2-^. The endergonic reduction of ferredoxin (E_o_’= - 500 to - 420 mV) with NADH (E_o_’= - 320 mV) is driven by reverse electron transport across the membrane which, in turn, is an energy-driven process. A membrane-bound V-type ATPase is present in the genome of *A. neopropionicum*, encoded by the genes *atpA/ntpA* (CLNEO_280), *atpB/ntpB* (CLNEO_290), *ntpC*, (CLNEO_260), *atpD/ntpD* (CLNEO_23400), *atpE* (CLNEO_250), *ntpG* (CLNEO_270), *ntpK* (CLNEO_240) and *ntpI* (CLNEO_23330). We theorise that ATP is hydrolysed in the ATPase to create a proton- or sodium-motive-force that is used by the Rnf complex to catalyse the reduction of ferredoxin. The production of Fd^2-^ is an energy costly process, the implications of which are addressed later in this section.

Pyruvate produced by the PFOR is subsequently reduced to lactate with NADH via D-lactate dehydrogenase (CLNEO_28010). We assumed NADPH is not used as electron carrier in this reaction, since lactate dehydrogenases have a strict specificity for NAD^+^/NADH [65, 66]. Lactate then enters the acrylate pathway, a cyclic chain of reactions involving the intermediates lactoyl-CoA, acryloyl-CoA and propionyl-CoA. The characteristic enzyme of this pathway is propionate-CoA:lactoyl-CoA transferase (Pct, EC 2.8.3.1), which exchanges the CoA moiety between propionyl-CoA and lactate, generating lactoyl-CoA and propionate as end product [67, 68]. Our first annotation of the genome of *A. neopropionicum* did not include Pct. However, an acetate CoA-transferase was present, encoded by the gene *ydiF* (CLNEO_17700), that shared 96 % identity with the purified and well-characterised Pct of *A. propionicum* [68]. Thus, we deduced that *ydiF* encodes for Pct in *A. neopropionicum* and included this reaction in the model. Lactoyl-CoA dehydratase (CLNEO_17710 and CLNEO_17720) catalyses the dehydration of lactoyl-CoA to acryloyl-CoA, which is subsequently reduced to propionyl-CoA by acryloyl-CoA reductase. Our genome annotation revealed that the acryloyl reductase of *A. neopropionicum* forms an enzymatic complex with an electron-transferring flavoprotein (EtfAB). The complex, hereafter named acryloyl-CoA reductase-EtfAB (Acr-EtfAB), is also present and has been well characterised in *A. propionicum* [69]. Three gene clusters predicted to encode for acryloyl-CoA reductase (*acrC*) or EtfAB (*acrA*,*acrB*) were found in the genome: (i) CLNEO_21740 (*acrC*), CLNEO_21750 (*acrB_1*) and CLNEO_21760 (*acrA*); (ii) CLNEO_26130 (*acdA_1*) and CLNEO_26120 (*acrB_2*); and (iii) CLNEO_29850 (*acdA_2*) and CLNEO_29840 (*acrB_3*). The *acdA_1* and *acdA_2* genes encode for acyl-CoA dehydrogenases that share low identity (46 and 54 %, respectively) with the acryloyl-CoA reductase encoded by *acrC*; thus, we assumed that the former two enzymes are not responsible for acryloyl-CoA reductase activity. The first cluster is the only complete one, composed of acryloyl-CoA reductase (*acrC*) and the A (*acrA*) and B (*acrB_1*) subunits of EtfAB. The proteins encoded by these three genes share an identity of 92.9 %, 89.7 % and 89.1 %, respectively, with their homologues from the Acr-EtfAB complex of *A. propionicum*. The Acr-EtfAB of *A. propionicum* is a non-bifurcating soluble enzyme that catalyses the irreversible reduction of acryloyl-CoA to propionyl-CoA with NADH via electron transfer to a flavin moiety and appears not to be involved in energy conservation [69, 70]. Given their high similarity, we deduced the same features apply to the Acr-EtfAB of *A. neopropionicum*. To our knowledge, this is the first time that the Acr-EtfAB complex is identified in this microorganism.

According to the theoretical stoichiometry, the fermentation of ethanol yields propionate and acetate in a 2:1 ratio (Eq. 1). However, this ratio is not observed in cultures of *A. neopropionicum*; rather, ethanol fermentation resulted in a $\approx$ 1.2:1 propionate to acetate ratio (Fig. 3 and Additional file 1: Table S2). We reasoned that the theoretical ratio cannot be achieved in *A. neopropionicum* due to energetic constraints of the cell, specifically, due to the requirement of Fd^2-^. Model simulations were performed to confirm this. The oxidation of three moles of ethanol generates six moles of NADH and three moles of acetyl-CoA. To fit the theoretical 2:1 propionate to acetate ratio, two moles of acetyl-CoA would have to be used in the reductive part of the metabolism, and one mole of acetyl-CoA should be invested in acetate, with the concomitant production of one mole of ATP via SLP. The synthesis of two moles of pyruvate from acetyl-CoA would require two moles of Fd^2-^, which is produced at the Rnf complex at the expense of ATP. However, the hydrolysis of one mole of ATP ($\Delta G^o$ = -32 kJ mol^-1^; [71]) could drive the reduction with NADH of no more than $\approx$ 1.3 moles of ferredoxin ($\Delta G^o$ = -25 kJ mol^-1^; [72]). Moreover, two other issues arise: i) even if this one mole of ATP would solely be invested in the reduction of ferredoxin, this would leave no net ATP for growth, and ii) such a scenario would result in excess reducing equivalents from ethanol oxidation that could not be recycled in the production of propionate. Our model predictions confirmed this inconsistencies and are in agreement with the hypothesis that the propionate to acetate 2:1 ratio cannot be achieved in *A. neopropionicum* during the fermentation of ethanol. Instead, cells must invest more than one mole of acetyl-CoA in acetate production to obtain net ATP to support growth. This leaves less than two moles of acetyl-CoA available for propionate production and, overall, a propionate to acetate ratio lower than the theoretical 2:1. The actual propionate to acetate ratio (close to 1.2:1, based on the fermentation balance) depends on how much Fd^2-^ can be produced per hydrolysed ATP, which in turn depends not only on the Gibbs free energies of ATP hydrolysis and ferredoxin reduction with NADH under physiological conditions but also on the coupling ratio of the ATPase (number of cations translocated per ATP hydrolised). While the Rnf complex can be assumed to translocate two cations per ferredoxin reduced/oxidised, the coupling ratio of the ATPase remains unknown for *A. neopropionicum*. Our model fitted with a coupling ratio of the ATPase of 3 to 3.5 H^+^ or Na^+^ translocated per ATP.

### Propanol and butyrate production pathways

**Fig. 6:**
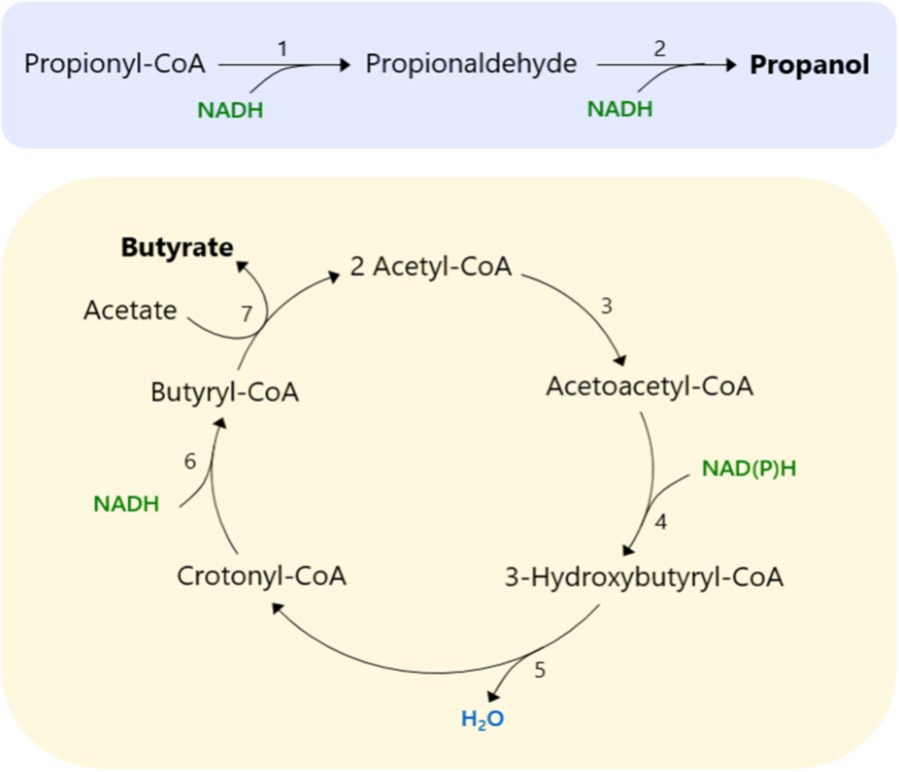
Putative pathways for the production of propanol (blue) and butyrate (orange) in *A. neopropionicum*. Numbers in reactions correspond to the following enzymes as annotated in the genome, and reaction ids in the model: 1 and 2, aldehyde-alcohol dehydrogenase (rxn09944_c0 and rxn01710_c0); 3, acetoacetyl-CoA thiolase (rxn00178_c0); 4, 3-oxoacyl reductase (rxn03861_c0); 5, 3-hydroxyacyl dehydratase (rxn03874_c0); 6, acryloyl-CoA reductase-EtfAB (rxn00868_c0) or acyl-CoA dehydrogenase-EtfAB; 7, propionate-CoA:lactoyl-CoA transferase (rxn00875_c0)

*A. neopropionicum* produces propanol and butyrate as minor products of the fermentation of several substrates (Fig. 2). Propanol is formed from propionyl-CoA via propionaldehyde in a two-step reductive conversion catalysed by AdhE (Fig. 6). Reduction of propionaldehyde could also be catalysed by NAD^+^-dependent alcohol dehydrogenases *adh* (CLNEO_16910) and *adhB* (CLNEO_00480).

Butyrate production in *A. neopropionicum* takes place via the acetyl-CoA pathway (Fig. 6). In this pathway, acetyl-CoA is first converted to butyryl-CoA, which eventually yields butyrate. Most enzymes of the pathway were either present in the genome, were assigned during the re-annotation or were identified through protein sequence alignment. Only one enzyme was not found: acetoacetyl-CoA thiolase (EC 2.3.1.9), which catalyses the condensation of two molecules of acetyl-CoA to form acetoacetyl-CoA. However, since the rest of genes of the pathway were identified (see Additional file 2), we added this reaction to the model during the gap-filling process.

A key enzyme of this pathway is the butyryl-CoA dehydrogenase/electron-transferring flavoprotein complex (Bcd-EtfAB). Bcd-EtfAB is an electron-bifurcating enzyme that couples the reduction of crotonyl-CoA to butyryl-CoA (E_o_’= -10 mV) by NADH to the endergonic reduction of Fd by NADH [63]. Our model predicts that, in *A. neopropionicum*, reduction of crotonyl-CoA could be catalysed by the Acr-EtfAB complex or by either of the two acyl-CoA dehydrogenases that cluster with subunits of the EtfAB complex (*acdA_1*-*acrB_2* and *acdA_2*-*acrB_3*). Among the three, the acyl-CoA dehydrogenase encoded by *acdA_2* showed the highest identity with the butyryl-CoA dehydrogenases (Bcd) of *C. acetobutylicum* and of *C. kluyveri* (64 and 63 %, respectively). It remains a question whether, in *A. neopropionicum*, the latter two complexes could be involved in the reduction of ferredoxin.

Two distinct routes have been described for the last step of the pathway, the conversion of butyryl-CoA to butyrate. The first route, identified in *C. acetobutylicum* [73], involves phosphate butyryltransferase (Ptb; EC 2.3.1.19) and butyrate kinase (Buk; EC 2.7.2.7) and yields ATP via SLP. The second route relies on butyryl-CoA:acetate CoA-transferase (But; EC 2.8.3.8). Co-occurrence of both pathways is rare among butyrate producers [74]. The genome of *A. neopropionicum* does not encode for Ptb nor Buk, yet our annotation initially assigned these activities to phosphate acetyltransferase (Pta) and acetate kinase (Ack). The Ack of *A. neopropionicum* is significantly similar to the well-characterised Buk (71 % identity) of *C. acetobutylicum*. We have considered this similarity to arise from the fact that the two enzymes belong to the same family, yet it has been established that they do not have the same function, since differences in the substrate binding site ultimately determine substrate specificity [75–77]. Thus, we assumed that Pta and Ack are not involved in butyrate production in *A. neopropionicum*. Instead, we hypothesise that butyrate production in *A. neopropionicum* takes place via butyryl-CoA:acetate CoA-transferase activity. Our model predicts that the propionate-CoA:lactoyl-CoA transferase (Pct) encoded by the gene *ydiF* catalyses this reaction. The Pct of *A. propionicum* exhibits broad substrate specificity for monocarboxylic acids, including butyrate, supporting the model prediction [68].

### Identification of the NADH-dependent reduced ferredoxin:NADP^+^ oxidoreductase (Nfn)

During the genome re-annotation and manual curation process, we identified the enzyme NADH-dependent reduced ferredoxin:NADP^+^ oxidoreductase (Nfn). Nfn is an iron-sulfur flavoprotein complex with electron-confurcating/bifurcating activity that reversibly catalyses the endergonic reduction of NADP^+^ by NADH coupled with the exergonic reduction of NADP^+^ by Fd^2-^ [78]. Nfn is composed of two subunits, NfnA and NfnB, whose coding genes were both found in the genome of *A. neopropionicum* under the locus tags CLNEO_00270 and CLNEO_00280, respectively. In the initial automatic annotation, these two genes were assigned to ferredoxin:NADP^+^ oxidoreductase and glutamate synthase, respectively. It has been reported that NfnA/B share sequence similarities with these two enzymes [78]. Upon manual inspection, we observed that the protein complex showed a significant identity (60 - 66 $\%$) with the Nfn complexes of *C. kluyveri* [79] and of *C. autoethanogenum* [80], which lead us to the re-assignation of the two proteins as NfnA and NfnB.

We used modelling to look into the role of the Nfn complex in the metabolism of *A. neopropionicum* during growth on ethanol. The model shows that the Nfn generates NADPH from NADH and Fd^2-^ for NADPH-dependent reactions of the cell. For instance, NADPH is required during butyrate production in the reduction of acetoacetyl-CoA to 3-hydroxybutyryl-CoA, a reaction catalysed by a NADPH-dependent 3-oxoacyl reductase. NADPH is also required in the biosynthesis of amino acids and biomass precursors. In our model, the Nfn complex does not function in the reverse direction, the production of Fd^2-^, during growth on ethanol; this would require NADPH, and ethanol oxidation is assumed to occur only via NAD^+^-dependent reactions.

### Fermentation of other carbon sources: the case of lactate

Besides ethanol, *A. neopropionicum* can grow on lactate, sugars and some pyruvate-derived amino acids (Table 2). The fermentation of these carbon sources proceeds with key differences compared to the fermentation of ethanol. To illustrate this with an example, we used the model to describe the case of lactate fermentation, since lactate is a typical substrate of propionate-producing bacteria and, in particular, of species that use the acrylate pathway [16, 67].

Lactate is metabolised in both oxidative and reductive reactions. In the oxidative branch, lactate is oxidised to pyruvate via lactate dehydrogenase, generating NADH. PFOR then catalyses the decarboxylation of pyruvate to acetyl-CoA and CO_2_, a reaction that generates Fd^2-^. The PFOR reaction is reversible; here, it functions in the opposite direction to what occurs with ethanol as substrate. This enables the utilisation of lactate, sugars and pyruvate-derived amino acids. This implies that, contrary to the fermentation of ethanol, the oxidation of these substrates generates directly Fd^2-^, which can contribute to energy conservation. Acetyl-CoA is used for acetate production via Pta and Ack, yielding ATP via SLP. In the reductive branch, lactate is converted to propionate via the reactions of the acrylate pathway. In this conversion, NADH is needed for the reduction of acryloyl-CoA to propionyl-CoA, but the amount of NADH obtained in the oxidation of lactate is insufficient. Our model predicts that additional NADH is produced in the Rnf complex. Opposite to the scenario with ethanol as substrate, here the Rnf catalyses the exergonic reduction of NAD^+^ with electrons from Fd^2-^. This reaction is coupled to the translocation of two cations across the membrane, generating an ion-motive force that can be used by the ATPase to produce ATP. Thus, in the fermentation of carbon sources other than ethanol, ATP is generated both by SLP via acetate production and by chemiosmosis driven by the oxidation of Fd^2-^.

## Discussion

In this study, we have presented iANEO_SB607, the first GEM of the propionate-producer *A. neopropionicum*. The overall Memote score of 72 % indicates the high quality of the model. The low score of gene annotation per database (33 %) was expected, since there were almost no available annotations of the genome of *A. neopropionicum* in public databases recognised by Memote. A limitation of the GEM is the lack of an organism-specific biomass composition and GAM/NGAM measurements. Our sensitivity analysis showed a maximum deviation of 10.8 % of the growth rate when varying the composition of biomass components or the GAM. NGAM has a more limited impact on growth rate predictions, given that it does not directly relate to the biomass synthesis reaction, still dedicated measurements of these parameters could furthe improve the predictive power of the model. Here, our focus has been on gaining insight into the metabolism of ethanol fermentation to propionate, which in this bacterium occurs via the acrylate pathway.

We have also addressed an important issue regarding the energetic metabolism of *A. neopropionicum*. During growth on ethanol, Fd^2-^ is required to reduce acetyl-CoA to pyruvate. In the earliest description of the metabolism of *A. neopropionicum*, authors suggested that the oxidation of acetaldehyde proceeded with ferredoxin as electron carrier, thus fulfilling this demand [17]. However, at the present time it is acknowledged that aldehyde dehydrogenases are NAD(P)-dependent enzymes [81], which invalidates that theory. Theoretically, the Acr-EtfAB complex could drive the reduction of Fd (E_o_’= - 500 to - 420 mV) with NADH (E_o_’= - 320 mV) via electron bifurcation, given the high reduction potential of the acryloyl-CoA/propionyl-CoA pair (E_o_’= + 70 mV). Yet, this complex appears not to be involved in the reduction of ferredoxin [69], most likely to prevent transient accumulation of the very reactive intermediate acryloyl-CoA [82, 83]. Instead, our model predicted that Fd^2-^ is produced in the Rnf complex, as previously reported for other anaerobes during growth on low-energy substrates [64]. The Rnf complex had been previously identified in the close relative *A. propionicum* [29]. Here, through the re-annotation an a thorough manual curation process, we identified all its subunits (*rnfA-E*, *rnfG*) and via modelling we verified its involvement in the metabolism of the cell.

Our annotation of the genome of *A. neopropionicum* revealed the presence of another key enzyme of the metabolism of anaerobes: the Nfn complex. Our model showed that the Nfn generates NADPH for NADPH-dependent reactions of the metabolism, which is essential during growth. Further investigation is needed to define the instances in which the Nfn operates in the reverse direction, bifurcating electrons from NADPH to produce Fd^2-^ and NADH. The directionality and role of Nfn will depend on the cofactor requirements of the cell.

Butyrate and propanol are produced by *A. neopropionicum* as minor products during the fermentation of several substrates (Fig. 2), probably as a means to dispose of excess reducing equivalents generated during substrate oxidation. Our model showed that propanol is produced from propionyl-CoA with propionaldehyde as intermediate via NAD^+^-dependent reactions, as Tholozan et al. suggested [17]. The butyrate production pathway had not been described yet in this microorganism and further research is needed to confirm whether Pct is indeed involved in this pathway as observed *in vitro* in *A. propionicum* [68] and *E. coli* K-12 [84].

Another aspect of the metabolism that we aimed to clarify was the ability of *A. neopropionicum* to produce and consume H_2_. In our batch cultivations on different substrates, H_2_ was not produced nor consumed, as previously reported [17]. Our results also confirm that neither the product profile nor the growth of ethanol-growing cultures of *A. neopropionicum* are affected by the presence of H_2_ (Additional file 1, Figure S3). This is an advantageous trait when considering this strain for its application in syngas-fermenting co-cultures, since syngas contains H_2_. Interestingly, H_2_ tolerance is manifested differently in functionally-related strains. While *P. propionicus* and *D. propionicus* both use the methylmalonyl pathway to metabolise ethanol, the first is not affected by the presence of H_2_ while the second is strongly inhibited by it [14].

The GEM iANEO_SB607 accurately reproduced observed growth phenotypes on typical substrates (ethanol, sugars, lactate and amino acids). For glucose and xylose, model predictions agree with our batch incubations that *A. neopropionicum* can utilize these sugars (Additional file 1: Table S2). These analyses solve contradictions in literature most likely attributable to differences in media compositions across studies [9, 11, 17, 85]. Our results also indicate that D-lactate, and not L-lactate, support growth of *A. neopropionicum*, as previously observed [17]. Yet, the latter authors reported lactate dehydrogenase activity in cell-free extracts with D-, L- and DL-lactate, and hypothesised the presence of a lactate racemase which is absent in our annotated genome. However, *A. neopropionicum* has both L- and D-lactate dehydrogenases, so it cannot be excluded that L-lactate is also metabolised, perhaps at a much slower rate [86].

dFBA simulations showed good agreement with the dynamics of ethanol (and ethanol plus acetate) fermentation by *A. neopropionicum* in batch cultivation (Fig. 3 and Fig. 4). With ethanol as substrate, the theoretical 2:1 molar ratio of propionate to acetate (Eq. 1) was not achieved; instead, this ratio was $\approx$ 1.2:1 (Fig. 3 and Additional file 1, Table S2), matching previous observations [9, 17]. The model helped clarify this aspect. During growth on ethanol, ATP is solely produced via SLP. Net ATP generation required to sustain growth and to drive ferredoxin reduction needed by PFOR appear as the main cause of the observed propionate to acetate ratio of 1.2:1. In addition, cells might favour acetate over propionate synthesis to prevent accumulation of acryloyl-CoA [67]. Finally, propanol production at the end of the fermentation, likely to halt further acidification of the environment, also contributes to decrease the propionate to acetate ratio observed in batch cultures.

Interestingly, we observed that a low acetate concentration (< 25 mM) or low acetate:ethanol ratio (< 1) at the start boosted the growth rate and propionate production rate of *A. neopropionicum* during growth on ethanol. However, despite higher rates, final biomass concentrations in batch cultivations were slightly lower in the presence of acetate (10 or 25 mM). Our model showed increase flux through acetate:CoA ligase (*acs*; EC 6.2.1.1) in the presence of acetate (10 mM). This reaction assimilates acetate consuming ATP, which would explain the lower biomass concentrations observed. Model predictions showed that, in this scenario, more acetyl-CoA is converted to pyruvate through PFOR, which is another energy-consuming step. We also observed a higher flux through butanoyl-CoA:acetate CoA-transferase (catalysed by Pct). Batch cultivation experiments did not show a noticeable increase in butyrate concentration when acetate was present, rather lower. Therefore, we hypothesise that, in vivo, most acetate consumed is assimilated via acetate:CoA ligase, as our model predicts, or via reverse direction of PTAr and ACKr, instead of Pct. This deviation to the model is likely due to the fact that biomass synthesis was set as maximization objective in dFBA which would be achieved by a higher flux of acetate towards butyrate instead of assimilating it, saving ATP.

Overall, this work shows the advantages of using a model-driven approach to gain insight into the metabolism of microorganisms. The new findings fill in knowledge gaps and unravel key metabolic features of *A. neopropionicum*. As a result, this study means a step forward to further exploit this species as a cell factory for propionate production in mono-culture or in co-cultivation from sustainable feedstocks, e.g., syngas, as recently stated by Moreira et al. [15]. Additionally, *A. neopropionicum* can act as an intermediate species to extend the range of products from propionate to longer odd-chain carboxylic acids.

## Conclusions

In this study, we have constructed iANEO_SB607, the first GEM of *A. neopropionicum*. Combining experimental data with a manual curation of the annotated genome and a comprehensive network reconstruction, we have gained insight into the central carbon and energetic metabolism of this microorganism. The model predicted the metabolic capabilities of *A. neopropionicum* with high accuracy, which allowed us to investigate with detail the enzymatic routes involved in the fermentation of ethanol to propionate. Our analysis showed that *A. neopropionicum* produces propionate via propionate-CoA:lactoyl-CoA transferase, the characteristic enzyme of the acrylate pathway. Our *in silico* analysis revealed, for the first time in this microorganism, the presence of the electron-bifurcating Nfn complex. This model provides the basis to explore the capabilities of *A. neopropionicum* as microbial platform for the production of propionate from dilute ethanol as substrate. While beyond the scope of this study, the construction of this model signifies a step closer towards the development of multi-species models that describe syngas-fermenting co-cultures comprised of acetogens with ethanol-consuming propionigenic bacteria. Follow-up studies that integrate, e.g., omics analyses with data from steady-state fermentations should help improve this GEM.

## Supplementary Information

**Additional file 1: Figure S1.** Sensitivity analysis of the biomass reaction. Effect of varying the composition of main biomass building blocks on the growth rate and product formation. Growth rate and product formation are represented as the difference between the values obtained when the new biomass reaction is defined as objective function and the values obtained when the original biomass synthesis reaction is defined as objective function.** Figure S2.** Effect of varying the growth-associated maintenance (GAM) of the biomass composition on the growth rate. The effect is represented as the difference between the values obtained when the new biomass reaction is defined as objective function and the values obtained when the original biomass synthesis reaction is defined as objective function.** Table S1.** Parameters used to simulate batch fermentations through dFBA. Column ‘Source’ indicates whether the parameter was constrained based on the experimental value (considering standard deviation error) or by model fitting.** Figure S3.** Effect of H2 on ethanol-growing cultures of Anaerotignum neopropionicum.** A)** Cell growth profiles, determined by optical density at 600 nm (OD600).** B)** End products and ethanol consumed at the end of batch fermentations. Error bars indicate the standard deviation of biological triplicates.** Table S2.** Fermentation balance of batch cultures of A. neopropionicum cultivated on different substrates. Note that CO2, present in the headspace of bottles, is consumed but not included in this table. iBut: isobutyrate; iVal: isovalerate. Traces are concentrations < 0.2 mM. The hyphen symbol indicates undetected products. ND indicates not determined.

**Additional file 2.** Genes and homologos of the acrylate and butyrate pathways found in other *Clostridium* species.

## Data Availability

The supplementary information and the data generated during the current study are available in the following https://gitlab.com/wurssb/Modelling/Anaerotignum_neopropionicum
